# Selective Laser Sintering 3D Printing of Carvedilol Tablets: Enhancing Dissolution Through Amorphization

**DOI:** 10.3390/pharmaceutics17010006

**Published:** 2024-12-24

**Authors:** Nikola Pešić, Branka Ivković, Tanja Barudžija, Branka Grujić, Svetlana Ibrić, Djordje Medarević

**Affiliations:** 1 Department of Pharmaceutical Technology and Cosmetology, Faculty of Pharmacy, University of Belgrade, Vojvode Stepe 450, 11221 Belgrade, Serbia; nikola.pesic@pharmacy.bg.ac.rs (N.P.);; 2 Department of Pharmaceutical Chemistry, Faculty of Pharmacy, University of Belgrade, Vojvode Stepe 450, 11221 Belgrade, Serbia; 3 Vinča Institute of Nuclear Sciences, National Institute of the Republic of Serbia, University of Belgrade, Mike Petrovića Alasa 12–14, 11351 Belgrade, Serbia; tbarudzija@vin.bg.ac.rs; 4 Galenika a.d., Batajnički drum bb., 11080 Belgrade, Serbia

**Keywords:** 3D printing, selective laser sintering, poorly soluble drugs, amorphous state, dissolution improvement

## Abstract

Background/Objectives: Selective laser sintering (SLS) is one of the most promising 3D printing techniques for pharmaceutical applications as it offers numerous advantages, such as suitability to work with already approved pharmaceutical excipients, the elimination of solvents, and the ability to produce fast-dissolving, porous dosage forms with high drug loading. When the powder mixture is exposed to elevated temperatures during SLS printing, the active ingredients can be converted from the crystalline to the amorphous state, which can be used as a strategy to improve the dissolution rate and bioavailability of poorly soluble drugs. This study investigates the potential application of SLS 3D printing for the fabrication of tablets containing the poorly soluble drug carvedilol with the aim of improving the dissolution rate of the drug by forming an amorphous form through the printing process. Methods: Using SLS 3D printing, eight tablet formulations were produced using two different powder mixtures and four combinations of experimental conditions, followed by physicochemical characterization and dissolution testing. Results: Physicochemical characterization revealed that at least partial amorphization of carvedilol occurred during the printing process. Although variations in process parameters were minimal, higher temperatures in combination with lower laser speeds appeared to facilitate a greater degree of amorphization. Ultimately, the partial conversion to the amorphous form significantly improved the dissolution of carvedilol compared to its pure crystalline form. Conclusions: Obtained results suggest that the SLS 3D printing technique can be effectively used to convert poorly water-soluble drugs to their amorphous state, thereby improving solubility and bioavailability.

## 1. Introduction

Three-dimensional (3D) printing is a type of additive manufacturing that is moving the pharmaceutical sector away from mass production of fixed-dose medicines towards the flexible manufacture of personalized medicines with doses or other properties tailored to the patient [1]. Three-dimensional printing enables the production of objects which would otherwise be impossible to produce with conventional manufacturing processes, by fabrication in a layer-by-layer manner, according to predefined model. This enables fast fabrication of small batches of dosage forms whose properties are adjusted to each patient [2,3,4]. Since the approval of Spritam^®^ as the first 3D-printed drug, the field of 3D printing has advanced rapidly, with groundbreaking research highlighting its numerous innovative applications. This progress has driven researchers to further explore and evaluate a broader range of 3D printing technologies [1].

Although numerous studies show the benefits of 3D printing in the fabrication of personalized dosage forms, implementation of this technique in the healthcare system is hampered by a lack of a specific regulatory framework for 3D-printed dosage forms. FDA approval of Spritam^®^ in 2015 represents an important milestone, which was achieved through the conventional procedure with the active involvement of the novel FDA Emerging Technology Team (ETT). In 2017, the FDA highlighted 3D printing as an important future technology for pharmaceutical manufacturing and issued guidance for its integration into the industry. Similarly, China’s Center for Drug Evaluation (CDE) recognized the potential of 3D printing for personalized drug delivery in 2019. Triastek’s MED 3D printing technology was approved by the FDA in 2020, further confirming the regulatory acceptance of 3D printing in drug production. However, despite these advancements, regulatory guidelines for 3D-printed pharmaceuticals are still lacking, and there is an urgent need for established standards as the technology continues to evolve. It is anticipated that, as the technology advances and further research is conducted, 3D printing will facilitate the establishment of comprehensive scientific standards in the pharmaceutical sector, bridging the gaps from theory to practice and from production to regulation [5].

Numerous different 3D printing techniques have been investigated for producing personalized medicines such as fused deposition modeling (FDM), binder and material jetting, semi-solid extrusion (SSE), stereolithography (SLA), and selective laser sintering (SLS) [6,7,8,9]. Selective laser sintering (SLS) represents an additive manufacturing technique which enables the building of objects directly from the powder material via the fusion of powder particles through heating induced by localized laser irradiation [1]. During the printing process, a specific pattern corresponding to the predefined model is drawn onto the surface of the powder bed by laser irradiation. The 3D object is built layer-by-layer, where once the first layer is completed, a roller distributes a new layer of powder on top of the previous one followed by laser irradiation [10]. A high resolution, the possibility of recycling the powder, and the absence of pre-processing are the main benefits of this technique [6]. As a solvent-free process, SLS offers fast production since there is no need for further processing steps for solvent removal [10,11]. SLS also offers the possibility to print tablets with different drug release properties. It is of particular importance that SLS 3D printing enables the fabrication of very porous, fast-dissolving dosage forms, such as orodispersible tablets, which are difficult to produce by other 3D printing techniques. Whether SLS 3D-printed tablets exhibit immediate or sustained release depends on the formulation composition, but also on the process parameters, such as laser scanning speed, laser power, and hatch spacing, as well as set temperature in the printing chamber and layer thickness [11,12].

In the study by Tabriz et al. (2023), SLS was employed for the design and fabrication of personalized carvedilol (CVD) dosage forms in three different strengths, all meeting the pharmacopoeial requirements for tablet hardness, friability, and drug release. The findings of this study revealed that the process parameters significantly influenced the characteristics of the produced tablets; specifically, adjusting the laser intensity impacted key tablet quality attributes, including hardness, friability, dissolution rate, and total amount of drug released [13]. Additionally, the printing process is also affected by the powder characteristics, such as particle size, shape, and flowability. Based on previous research, Basit et al. (2017) pointed out that the recommendation for the successful SLS 3D printing process is that the particles should have good flow properties, and their size should be in the range from 58 to 180 μm [14,15]. The use of photoabsorbers is also necessary for most powder mixtures when printing is performed by printers with lasers that operate in the visible light region [16,17]. Possible drug or excipient degradation induced by laser irradiation and/or heatingin the printing chamber are the main disadvantages of this technique [18] Despite this drawback, SLS represents a promising method of printing porous dosage forms without using a liquid binder [19,20]

Amorphization of the crystal forms is considered a promising approach to significantly enhance oral bioavailability of poorly soluble drugs due to the solubility advantage of a disordered amorphous state [21]. Unfortunately, amorphous forms are thermodynamically unstable and tend to convert into a lower energy crystalline state. However, different polymeric excipients have been successfully used as stabilizers of amorphous states, which is commonly ascribed to a specific intermolecular interaction and reducing molecular mobility in the amorphous state [21,22]. Potential applications of SLS in the fabrication of different dosage forms are still being investigated due to a lack of information on the process and material attributes required to obtain dosage forms that meet common quality requirements. The application of the SLS 3D printing technique for the amorphization of poorly soluble drugs is particularly unexplored.

Carvedilol (CAR) is a non-selective beta-adrenergic and alpha-1 adrenergic receptor antagonist, commonly prescribed for the management of mild to moderate congestive heart failure and hypertension. It is classified as a Biopharmaceutics Classification System (BCS) Class II compound, characterized by its limited aqueous solubility and high permeability [23]. Pharmacokinetics and the therapeutic response to carvedilol are affected by the genotype of the patient [24], which indicates a need for the formulation of personalized dosage forms with this drug. Therefore, this study aimed to investigate the potential application of SLS 3D printing for the fabrication of tablets with the poorly soluble drug carvedilol, with the aim of improving the drug dissolution rate through the formation of an amorphous form induced by the printing process.

## 2. Materials and Methods

### 2.1. Materials

The following substances were used in the experimental work: carvedilol (Hemofarm AD, Vršac, Serbia) as a model substance; while polyvinyl alcohol (PVA)-Parteck^®^ MXP (Merck, Darmstadt, Germany), mannitol-Parteck^®^ M 200 (Merck, Darmstadt, Germany), talc (Merck, Darmstadt, Germany), and Candurin^®^ Gold Sheen (Merck, Darmstadr, Germany) were used as excipients in tablet formulations.

### 2.2. Methods

#### 2.2.1. Characterization of Powder Blends

Powder Flow Properties

For both prepared powder blends, the flowability was tested by determining the bulk and tapped density and calculating the Hausner ratio and the compressibility index (Carr index, CI). The StaV 2003 volumeter (J. Engelsmann AG, Ludwigshafen, Germany) was used to determine the tapped density, and the Hausner ratio and compressibility index were calculated using Equations (1) and (2) presented below. The flowability of the powders was estimated according to the descriptive terms given in the current European Pharmacopoeia–Ph. Eur. 11.0 (Chapter 2.9.36. Powder flow) [25].

Hausner ratio = tapped density/bulk density(1)

Compressibility index (CI, %) = 100 × (tapped density − bulk density)/tapped density(2)

#### 2.2.2. Tablet Formulation

Within this research, eight types of tablet formulations (where the term formulation refers to a defined composition and process parameters) were obtained using two mixtures of powders and four combinations of printing parameters. The composition of used powder blends is given in Table 1. The formulation components were mixed in a powder mixer (Pharmalabor, Canosa di Puglia, Italy) at a speed of 60 rpm for 10 min. Before printing, both powder blends were sieved through a sieve with an aperture size of 180 µm.

#### 2.2.3. Preparation of Tablets Using SLS 3D Printing Process

Tablet formulations were printed using an SLS 3D printer, the Sintratec Kit (Sintratec, Brugg, Switzerland), using the process parameters given in Table 2. Prior to printing, a 3D model of the tablet with a diameter of 9.00 mm and a thickness of 4.00 mm was designed using Autodesk Fusion 360 software, version 2.0.8809 (Autodesk Inc., San Francisco, CA, USA). The 3D model, saved in .stl format, was then imported into the Sintratec Central software (Sintratec, Brugg, Switzerland). After importing, the printing parameters were configured, and the powder was loaded into the printer chamber.

The process parameters were optimized based on the physical properties of the mixture components and the results of the preliminary experiments. The initial phase of the research was conducted under lower temperature conditions (Surface Temperature: 50 °C, Chamber Temperature: 40 °C). These temperature settings were gradually increased to achieve tablets with enhanced mechanical characteristics and to facilitate the amorphization of the active substance. For both powder blends, the tablets exhibited optimal mechanical properties when the surface temperature reached 80 °C and 90 °C, and the chamber temperature was set at 70 °C and 80 °C. Consequently, these temperature parameters were selected for the SLS 3D printing process.

When evaluating the effect of laser speed, initial tests were conducted using higher speeds (180 mm/s). However, under these conditions, the tablets were either unprintable or exhibited poor mechanical properties. Consequently, lower laser speeds (50 and 60 mm/s) were implemented. For each batch, 160 g of powder was used. Six tablets were printed in one printing run.

#### 2.2.4. Characterization of the Tablets

Tablet Mass and Dimensions

The weight of the 3D-printed tablets was measured on a Sartorius BP 210D analytical balance (Sartorius, Göttingen, Germany). The mass variation test was performed on a sample of 20 randomly selected tablets obtained from each combination of experimental conditions and by calculating the average mass.

Tablet dimensions (diameter and thickness) were measured using a digital caliper (Vogel, Kevelaer, Germany) on the sample of 20 tablets obtained from each combination of experimental conditions. Results are expressed as the mean value with the standard deviation.

Drug Assay

Twenty tablets were finely powdered using a mortar and pestle. Each sample, equivalent to the average tablet mass, was accurately weighed and dissolved in 10 mL of methanol in the volumetric flask. The solution was subjected to ultrasonic agitation for 15 min to ensure dissolution, cooled to room temperature, and subsequently filtered through a 0.45 µm membrane filter. Quantification of dissolved carvedilol was conducted using a high-performance liquid chromatography (HPLC) method on a Dionex Ultimate 3000 system (Thermo Scientific, Waltham, MA, USA). For the analysis, a C18 column (250 × 4.6 mm, 5 µm) was utilized with a mobile phase comprising a 60:40 (v/v) mixture of 10 mM KH_2_PO_4_ buffer (pH = 3) and acetonitrile. The method employed a flow rate of 1.2 mL/min, an injection volume of 20 µL, and a detection wavelength of 240 nm. Measurements were performed in triplicate. The drug assay outcome was expressed as the amount of CVD (mg/tablet) and as a percentage of the theoretical drug content with standard deviations.

Uniformity of the Content

A single tablet was placed in 10 mL volumetric flasks, methanol was added, and the flasks were subjected to an ultrasonic bath for 15 min. The resulting solution was then allowed to cool to room temperature and filtered. We transferred 1 mL of this solution to a 10 mL volumetric flask and diluted it with the mobile phase up to the calibration mark. Testing was performed on 10 tablets of each formulation. The amount of CVD was determined using the HPLC method as described in the drug assay procedure.

Tablet Mechanical Resistance

The breaking force of the tablets was tested on an Erweka TBH 125D tablet hardness tester (Erweka, Langen, Germany). The force required to break the tablet was measured on a sample of 3 tablets obtained for each combination of process parameters separately. Based on the obtained values, the tensile strength was calculated using Equation (3):

σ = 2F/πRt(3)

σ—tensile strength, F—the breaking force of the tablet, R—tablet diameter, and t—tablet thickness.

Tablet Disintegration Testing

Tablet disintegration testing was performed using an Erweka ZT 54 disintegration tester (Erweka, Langen, Germany) in distilled water as a medium at a temperature of 37 ± 2 °C. Three tablets obtained from each combination of experimental conditions were tested until the last tablet disintegrate, or up to 30 min.

Powder X-ray Diffraction (PXRD)

X-ray powder diffraction was used to estimate the crystallinity of carvedilol in SLS 3D-printed tablets. The analyses were performed using a Philips PW-1050 diffractometer (Philips, Amsterdam, The Netherlands). The samples were tested in the range of 2*θ* angles from 5 to 50° using a CuKα (λ = 1.5418 Å) radiation source, with a step size of 0.02°, a dwell time of 1 s per step at a voltage of 40 kV, and a current of 30 mA.

Differential Scanning Calorimetry (DSC)

Differential scanning calorimetry was used to evaluate the physical state of carvedilol and all excipients used in 3D-printed tablets. A DSC1 Mettler Toledo (Mettler Toledo, Greifensee, Switzerland) instrument was used for analysis. Samples of powdered tablets (5–10 mg) were precisely weighed in 40 μL aluminum pans with perforated lids and then subjected to a heating program in the temperature range of 25–300 °C. The analyses were performed with a heating rate of 10 °C/min in a stream of nitrogen, at a flow rate of 50 mL/min. The obtained data were analyzed with the help of the software package STARe Software v12.10 (Mettler Toledo, Giessen, Germany).

Fourier Transform Infrared (FT-IR) Spectroscopy

FT-IR spectroscopy was used to detect potential intermolecular interactions between carvedilol and other components of the printed tablets. A Nicolet iS10 instrument (Thermo Fisher Scientific, Waltham, MA, USA) equipped with an ATR system (Smart iTR, Thermo Waltham Fisher Scientific, USA) with a Zn-Se lens was used to record FT-IR spectra of crushed tablets, pure carvedilol, and individual excipients. The spectra of the examined samples were recorded in the interval from 4000 to 650 cm^−1^, with a resolution of 2 cm^−1^, whereby 16 scans were performed for each spectrum.

Dissolution Testing

The dissolution rate of carvedilol from 3D-printed tablets was tested using the ERWEKA DT 600 mini paddle apparatus (Erweka, Lagnen, Germany). In order to achieve supersaturation of the active substance, non-sink conditions were used. Phosphate buffer (pH = 6.8) in a volume of 100 mL was used as the medium. The rotation speed of the mini paddles was set to 50 rpm and the working temperature was set to 37 ± 0.5 °C. Then, 2 mL of the solution was withdrawn at appropriate time intervals (5, 10, 15, 20, 30, 45, 60, 90, 120, 150, 180, 240, 360, 480 min) and immediately replaced with the same amount of fresh medium. All collected samples were filtered through a membrane filter with a diameter of 0.45 µm. The amount of dissolved carvedilol was determined by the HPLC method using a Dionex Ultimate 3000 (Thermo Scientific, Waltham, MA, USA) HPLC system under the following experimental conditions: C18 column (250 × 4.6 mm, 5 µm), mobile phase consisting of 60:40 (v/v) mixture of 10 mM KH_2_PO_4_ (pH 3) and acetonitrile, a flow rate of 1.2 mL/min, injection volume of 20 µL, and detection wavelength of 240 nm. The test was performed in triplicate.

## 3. Results and Discussion

### 3.1. Powder Flow Properties

The results of the flowability testing of the powder mixtures used for printing of the tablets are given in Table 3. The measured flowability values fall within the acceptable range defined by the descriptive criteria of Ph. Eur. 11.0, though they are near the threshold for poor flowability. Ensuring optimal flowability is crucial for achieving a uniform powder layer distribution and producing tablets with consistent characteristics. However, in our study, tablets meeting standard quality requirements were successfully produced, suggesting that although flowability is a significant factor in SLS 3D printing of tablets, good powder flowability may not be required in all cases.

### 3.2. Visual Appearance of the Tablets

**Figure 1:**
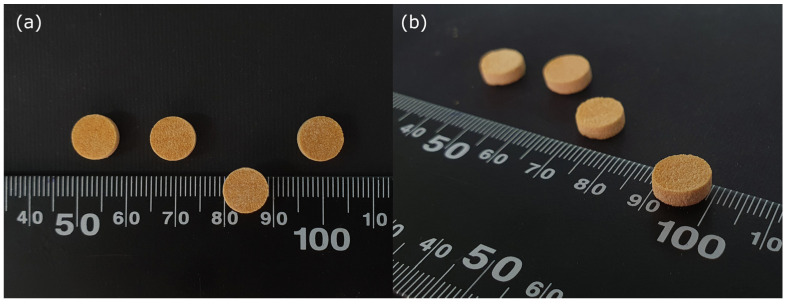
SLS printed tablets of powder blend 1 (**a**) and powder blend 2 (**b**) under varying printing conditions (surface temperature (°C)/chamber temperature (°C)/laser speed (mm/s) from left to right: 80/70/60, 80/70/50, 90/80/60, 90/80/50).

**Figure 2:**
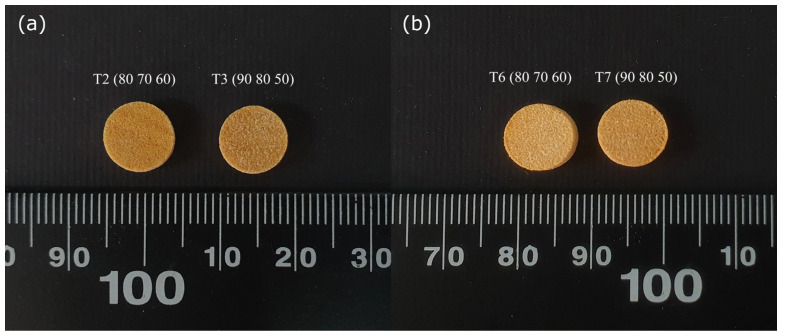
Tablets of powder blend 1 (**a**) and powder blend 2 (**b**) produced under the following printing conditions: surface temperature (°C)/chamber temperature (°C)/laser speed (mm/s) of 90 °C/80 °C/50 mm/s (right side in (**a**,**b**)) and 80 °C/70 °C/60 mm/s (left side in (**a**,**b**)).

Figure 1 displays the SLS 3D-printed tablets obtained from powder blends PB1 (Figure 1a) and PB2 (Figure 1b) produced under four different experimental conditions. Visually, it is challenging to distinguish between the tablets printed under different experimental conditions. However, when comparing the extremes of the printing conditions, as shown in Figure 2a for tablets obtained from powder blend 1 (T1–T4) and Figure 2b for tablets obtained from powder blend 2 (T5–T8), a noticeable difference can be observed. Tablets printed at a laser speed of 50 mm/s with a surface-layer temperature of 90 °C and a chamber temperature of 80 °C (right side of Figure 2a,b) are more resistant to stress during handling and less porous, resulting in slightly smaller dimensions compared to those printed at a laser speed of 60 mm/s under temperature conditions of 80 °C and 70 °C (left side of Figure 2a,b).

### 3.3. Tablet Mass and Dimensions

Table 4 shows the mass, diameter, and thickness of the tablets. On the basis of the obtained results (Table 5), it was concluded that the tablets prepared from both powder blends under all the four experimental conditions fulfill the pharmacopoeial (Ph. Eur. 11.0) requirements in terms of mass variation [25]. Previous studies have demonstrated that chamber temperature positively influences the mass of the printed tablets; as the chamber temperature increases, tablets with greater mass are produced. In contrast, laser scanning speed has an inverse effect. Tablets printed at higher laser speeds tend to have lower masses compared to those printed at lower speeds. At lower laser scanning speeds, the powder particles are exposed to the laser for a longer period, allowing them to absorb more energy during the sintering process. This, combined with higher chamber temperatures, leads to the melting and fusion of the polymer components in the powder mixture, reducing the void space between particles [26]. This phenomenon can also account for the results observed in our study. Tablets produced under higher temperature conditions and lower laser scanning speeds were less porous, resulting in a higher density, greater mass, and smaller dimensions. These findings are consistent with those reported by Fina et al. (2018), who found that tablets printed at higher laser scanning speeds are more porous, which corresponds with their lower mass [19].

### 3.4. Drug Assay and Uniformity of the Content

The drug assay demonstrated that the active substance remained stable without any degradation during the 3D printing process, despite the use of a high powder bed temperature. The measured values were within the range of 90–110% of the theoretical content of the active substance (Table 5), which falls within the acceptable criterion in the The United States Pharmacopeia monograph for carvedilol tablets [27].

In the study by Fina et al., paracetamol–polymer blends with loadings of 5%, 20%, and 35% were processed at various laser intensities to assess potential drug degradation with increasing intensity. The printed solid oral dosage forms revealed that a significant portion of paracetamol remained in its amorphous state, without degradation, even when printed at high laser intensities [10].

The content uniformity of the carvedilol SLS 3D-printed tablets was within acceptable criteria, demonstrating that the SLS 3D printing technique reliably produces tablets with uniform contents of active pharmaceutical ingredients.

### 3.5. Tablet Mechanical Resistance

Process parameters such as laser speed and chamber temperature significantly affect the hardness of tablets produced by SLS 3D printing. Previous studies have demonstrated that tablets printed at higher chamber temperatures have greater hardness and density due to an increased degree of sintering at elevated temperatures. Conversely, higher laser speeds result in tablets with lower hardness, as the shorter laser irradiation time reduces energy absorption by the powder mixture [26].

Tablets from both powder blends exhibited good handling properties and did not break easily during manipulation. Table 6 presents the hardness and tensile strength values for tablets produced from powder blends 1 and 2. For powder blend 1, when comparing tablets produced at the same temperatures, but different laser speeds, slightly higher hardness was achieved at lower speeds, as expected (T1 > T2 and T3 > T4). It was also shown that tablets printed at higher temperatures displayed greater hardness (T3 > T1 and T4 > T2). Similar results were observed for tablets obtained from powder blend 2, with tablet hardness values ranging from 55.8 N to 70.8 N and tensile strength values from 0.97 MPa to 1.28 MPa. Tablets produced at a laser speed of 50 mm/s and chamber temperature of 80 °C or 90 °C exhibited the highest hardness values. This can be attributed to the reduction in laser speed and the increase in chamber temperature, which together yield less porous tablets with stronger interparticle bonds, making them more resistant to breakage. Conversely, tablet T6 (70 80 60) was the most porous and exhibited the lowest hardness. These findings are consistent with those reported by Fina et al. (2018), where tablets prepared at higher laser scanning speeds demonstrated lower breaking force due to a reduced degree of sintering and fewer bonds formed between powder particles [19].

### 3.6. Disintegration of Tablets

The disintegration test results for tablets obtained from powder blends 1 and 2 are presented in Table 7. When comparing tablets produced under the same temperature conditions, but at different laser speeds, the disintegration time was slightly longer for tablets printed at lower laser speeds. Similarly, tablets produced at higher temperature settings showed longer disintegration times. However, given the relatively small variations in temperature and laser speed used in this study, the differences in disintegration times across different experimental conditions were almost negligible.

An increase in laser speed was found to correlate with increased tablet porosity [6]. For more porous tablets, a shorter disintegration time is expected, as it is assumed that the medium penetrates the tablet matrix more easily, facilitating quicker disintegration.

When comparing the disintegration times of both formulations (powder blends), it is evident that tablets from powder blend 2 disintegrated faster. This can be attributed to the addition of mannitol, which dissolves faster, leaving channels within the tablet structure, and further facilitates water penetration into the tablet [28].

### 3.7. Powder X-Ray Diffraction (PXRD)

**Figure 3:**
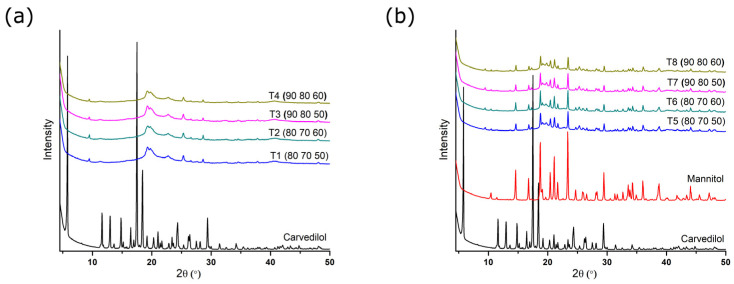
Diffractograms of carvedilol and powdered tablets obtained from powder blend 1 (**a**) and powder blend 2 (**b**) (printing parameters are given in the brackets in the following order: surface temperature (°C), chamber temperature (°C), and laser speed (mm/s)).

X-ray diffraction was performed to assess the degree of crystallinity of the active substance after the printing process. Figure 3 presents the diffractograms of the pure carvedilol and tablets obtained from powder blend 1 (Figure 3a) and powder blend 2 (Figure 3b). Pure carvedilol shows characteristic intense and sharp peaks at 5.8°, 11.6°, 13.0°, 14.8°, 17.5°, 18.45°, and 24.35° 2θ, indicating the crystalline nature of this active substance. The positions of the peaks agree with those previously reported for carvedilol polymorphic form II [29,30,31]. From the diffractograms of powdered tablets obtained by printing of powder blend 1, it can be observed that the characteristic peaks of carvedilol have almost disappeared, indicating its partial amorphization. However, since the peaks have not completely disappeared, it cannot be said that complete amorphization has occurred.

Similar results were obtained for tablets produced from powder blend 2 (T5–T8). The main peaks of carvedilol almost disappeared, indicating a huge decrease in the crystallinity. Raw mannitol exhibits sharp peaks at 10.4, 14.6, 16.75, 18.75, 20.4, 23.35, and 29.45° 2θ, which comply with diffraction patterns of its most stable β polymorphic form [32,33]. Most of the peaks that appear on the diffractograms of formulations T5-T8 originate from the mannitol, which retained its crystalline nature after being subjected to the SLS printing process.

In order to compare the influences of different printing parameters, the degree of crystallinity was calculated for carvedilol, mannitol, and all obtained printed tablets by an integration method that uses a straight background line and compares the area under the entire diffractogram with the area under the crystalline peaks using the following Equation (4):

Crystallinity = (area under the crystalline peaks)/(area under all peaks)(4)

The calculated degree of crystallinity values (Table 8) show the presence of residual crystallinity in all samples, which mostly originates from the excipients in tablet formulations.

Recently, Tabriz et al. (2023) confirmed the amorphization of carvedilol using SLS 3D printing technology. In that study, tablets were printed from a formulation that contained a mixture of carvedilol and Kollidon^®^ VA64 using a SnowWhite SLS printer (SHAREBOT, Nibionno, Italy) equipped with a 14 W CO_2_ laser. The temperature was set at 90 °C, as was the case in this research. The characteristic diffraction peaks of the carvedilol within the Kollidon VA64 physical blend showed that the drug was in a crystalline form prior to printing. However, the X-ray diffractograms of carvedilol from SLS 3D-printed tablets at different laser intensities showed haloes, indicating conversion of carvedilol to the amorphous state [13].

Madžarević et al. also reported the transformation of an API into an amorphous state as a result of SLS 3D printing. The findings suggested that for the polymer and drug used in this study, the SLS printing of solid dosage forms may be able to produce an amorphous solid dispersion, increasing the solubility of the API [34]. It is believed that amorphization of an active substance could be induced by an elevated temperature and laser irradiation during the printing process.

### 3.8. Differential Scanning Calorimetry (DSC)

**Figure 4:**
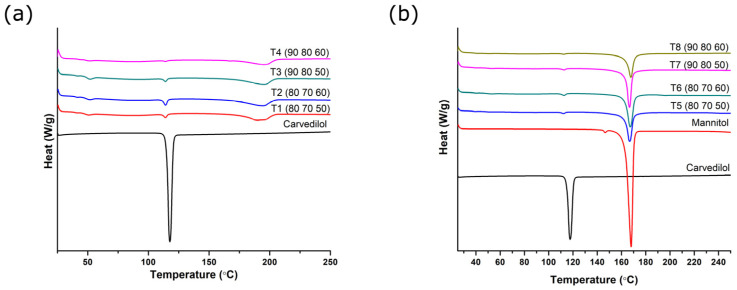
Thermograms of carvedilol and powdered tablets obtained from powder blend 1 (**a**), and powder blend 2 (**b**) (printing parameters are given in the brackets in the following order: surface temperature (°C), chamber temperature (°C), and laser speed (mm/s)).

In order to investigate the thermal behavior of substances, DSC analysis was performed. Figure 4 shows the thermograms of the active substance carvedilol and tablets obtained from powder blend 1 (Figure 4a) and powder blend 2 (Figure 4b). Carvedilol showed a sharp endothermic peak at 117.67 °C, which corresponds to the melting temperature of polymorphic form II [35].

In the case of both powder blends printed in the four different experimental conditions, very small carvedilol melting peaks are observed, so it is concluded that during printing, under the influence of temperature and laser energy, the largest portion of carvedilol in the tablet is converted to the amorphous state. If the experimental conditions under which the tablets were prepared are compared, lower melting enthalpies are observed when higher temperature conditions were used—the temperature of the surface layer as 90 °C and the temperature of the chamber as 80 °C—which indicates a higher degree of amorphization. On the other hand, carvedilol melting peaks are more pronounced at lower temperatures of the surface layer and printing chamber—80 °C and 70 °C, respectively. Therefore, it can be concluded that exposure to higher temperatures during SLS printing facilitates amorphization of the drug.

In the case of tablets obtained from powder blend 1 (Figure 4a), the broad peak at ~200 °C is related to polyvinyl alcohol, which occurs due to several phenomena such as evaporation of water from polymers and the glass transition [36]. In the case of tablets obtained from powder blend 2 (Figure 4b), these peaks are absent as the amount of PVA was reduced and replaced with mannitol compared to tablets obtained from powder blend 1. The pronounced endothermic peak in tablets obtained from powder blend 2, at 167.83 °C, is related to the melting of mannitol, and this peak remains at the same position in the thermograms of all printed formulations.

### 3.9. Fourier Transform Infrared (FT-IR) Spectroscopy

**Figure 5:**
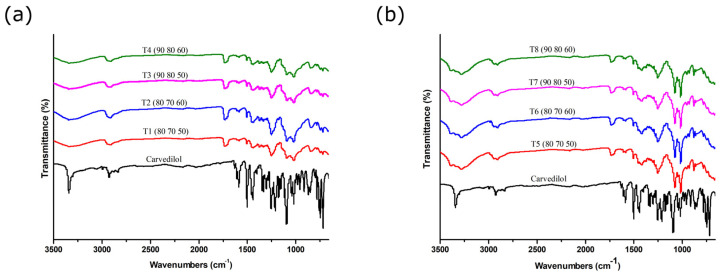
FT-IR spectra of carvedilol and powdered tablets obtained from powder blend 1 (**a**) and powder blend 2 (**b**) (printing parameters are given in the brackets in the following order: surface temperature (°C), chamber temperature (°C), and laser speed (mm/s)).

FT-IR spectroscopy was performed to investigate potential interactions between the active substance carvedilol and excipients in the formulations. Figure 5 shows the FT-IR spectra of carvedilol and tablets obtained from powder blends 1 and 2. Pure carvedilol showed characteristic absorption bands at 3341 cm^−1^ (N–H and O–H stretching vibrations), 2924 cm^−1^ (C–H aliphatic stretching vibrations), 1589 cm^−1^ (N–H bending vibrations), 1500 cm^−1^ (C–C aromatic stretching), and 1252 cm^−1^ (C–N stretching vibrations), which is in agreement with spectra available in the literature [31,37]. Due to differences in hydrogen bonding, absorption bands characteristic of N-H and O-H stretching vibrations are commonly used to distinguish carvedilol polymorphs. The absorption band is positioned at 3451 cm^−1^ in the case of form I, while in the case of form II, the same absorption band is positioned at 3345 cm^−1^ [38]. This confirmed the result of PXRD analysis, i.e., that carvedilol used in this study was polymorphic form II.

Changes in the carvedilol absorption bands positioned at 3341 cm^−1^ and between 1650 and 1550 cm^−1^ observed in the spectra of powdered SLS printed tablets indicated the presence of intermolecular interactions between carvedilol and the PVA polymer. Similar interactions were also observed in the case of co-amorphous systems of carvedilol and amino acids [39]. The observed interactions may facilitate the amorphization of carvedilol and further contribute to the stabilization of the amorphous form.

When analyzing FT-IR spectra of tablets obtained from powder blend 2 containing mannitol, it could be concluded that there were no detectable interactions between functional groups of carvedilol and mannitol, due to the absence of shifting of carvedilol absorption bands.

### 3.10. Dissolution Testing

**Figure 6:**
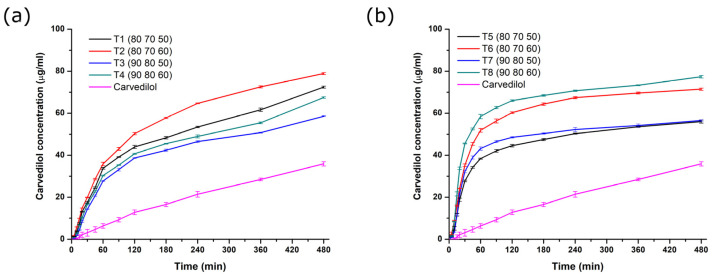
In vitro release profiles of carvedilol from printed tablets from powder blend 1 (**a**) and powder blend 2 (**b**) in comparison with pure active substance (printing parameters are given in the brackets in the following order: surface temperature (°C), chamber temperature (°C), and laser speed (mm/s)).

Figure 6 presents dissolution profiles of pure carvedilol and SLS 3D-printed tablets at different time points. It is clear to see that the dissolution rate of carvedilol from all tablets is improved relative to the pure carvedilol. The amorphous form of the active substance, which is at least partially generated by SLS 3D printing, has higher solubility, and thus a higher dissolution rate compared to the crystalline form [40,41]. Application of the SLS 3D printing technique for the amorphization of poorly soluble drug was also reported previously by Davis et al. Thanks to an optimum combination of the powder flow properties and SLS 3D printing parameters, tablets containing ritonavir-copovidone amorphous solid dispersions were successfully developed. This study confirmed that SLS 3D printing can be used as a single-step platform for creating amorphous solid dispersion-based pharmaceutical dosage forms with a solubility advantage [42].

In previous studies, it has been shown that drug release from SLS 3D-printed drug products can be controlled by varying the drug load, excipient type and amount, tablet geometry, and laser scanning speed [43].

Previous research has shown faster drug release from highly porous printed tablets due to the larger surface area available for contact with the medium, thus enabling its faster and deeper penetration inside the tablets, which increases the dissolution rate of the active substance. It was shown that by increasing the temperature of the chamber, the melting is intensified, and the porosity is lowered, which leads to a slower release of the API. On the other hand, when increasing the laser speed, the porosity of the tablets increases, which consequently leads to a faster release of the active substance [24]. Fina et al. (2018) showed that using a lower laser speed produced dosage forms with an increased density and reduced porosity than those obtained using a higher laser speed. This had a direct impact on the dissolution profiles of the active substance, so with a higher laser scanning speed, an accelerated release of the active substance was achieved [19].

A comparison of the carvedilol release rates from tablets made under different experimental conditions shows that the highest amount of drug was released from tablets made at lower printing temperature conditions (80 °C temperature of the surface layer and 70 °C chamber temperature) and at a higher laser speed (60 mm/s), which is explained by the fact that the tablets are more porous and the substance is released faster when in contact with the medium. On the other hand, tablets made at higher temperature conditions and at a slower laser speed are more compact, the distribution between the particles is denser, and the active substance is released more slowly. It can be seen from Figure 6 that carvedilol dissolution from tablets obtained from powder blend 1 (T1–T4) is significantly higher compared to pure carvedilol, which confirms the results of previous tests showing that amorphization of the active substance has occurred.

Even higher carvedilol concentrations were achieved from tablets obtained from powder blend 2 (T5–T8). This can be ascribed to the presence of mannitol (Parteck^®^ M 200), which, as a functional excipient, enables rapid disintegration and release of the API from the formulations [25].

The previously demonstrated amorphization certainly contributed to the improvement in carvedilol release from tablets compared to pure carvedilol. When comparing carvedilol release profiles from tablets made in different experimental conditions, it was shown that the laser speed has an important influence, where tablets printed at a laser speed of 60 mm/s released a higher amount of carvedilol, which is explained by their higher porosity.

Temperature effects, at the same laser speed, are different for tablets obtained from powder blend 1 (T1–T4) compared to tablets obtained from powder blend 2 (T5–T8). In the case of tablets obtained from powder blend 1, tablets produced at lower temperature conditions (90 °C temperature of the surface layer and 80 °C chamber temperature) showed faster drug dissolution, which can be explained by the more porous structure due to lower interparticle bonds. However, in the case of formulations containing mannitol, the highest carvedilol concentration in the dissolution medium was achieved from formulation T8, which was printed at a higher temperature and a lower laser scanning speed. Despite the higher temperature and lower laser scanning speed contributing to the formation of stronger tablets due to more intensive bonding between powder particles, this combination of process parameters facilitates drug amorphization, which may explain the higher amount of carvedilol dissolved from formulation T8. In this case, the negative effect of the higher temperature and lower laser scanning speed on drug release is prevented by the presence of highly soluble mannitol, which facilitates tablet disintegration and drug release.

## 4. Conclusions

This study demonstrated that tablets can be successfully produced using SLS 3D printing to enhance the dissolution rate of carvedilol. By employing the SLS 3D printing technique, eight tablet variants were created using two different powder mixtures and four combinations of experimental conditions. Physicochemical characterization revealed that at least partial amorphization of carvedilol occurred during the printing process. Although variations in process parameters were minimal, higher temperatures combined with lower laser speeds appeared to facilitate a greater degree of amorphization. Ultimately, the partial conversion to the amorphous form significantly improved the dissolution profile of carvedilol compared to its pure crystalline form. These findings suggest that the SLS 3D printing technique can be effectively used to convert poorly water-soluble drugs into their amorphous state, enhancing solubility and bioavailability.

## Figures and Tables

**Table 1:** Qualitative and quantitative compositions of the prepared powder blends (PBs).

		Quantity (%)
**PB 1**	Carvedilol	10
	Polyvinyl alcohol (PVA)	85
	Talc	2
	Candurin^®^ Gold Sheen	3
**PB 2**	Carvedilol	10
	Polyvinyl alcohol (PVA)	55
	Mannitol	30
	Talc	2
	Candurin^®^ Gold Sheen	3

**Table 2:** Printing parameters for different tablet formulations prepared from PB1 and PB2 (all formulations were printed with layer height set at 180 µm).

Tablet	Surface Temperature (°C)	Chamber Temperature (°C)	Laser Speed (mm/s)	Powder Blend Used in Formulation
T1	80	70	50	PB 1
T2	80	70	60	
T3	90	80	50	
T4	90	80	60	
T5	80	70	50	PB 2
T6	80	70	60	
T7	90	80	50	
T8	90	80	60	

**Table 3:** Flowability of the tested powder blends.

Powder Blend	Hausner Ratio	Compressibility Index (*CI*)	Flowability
PB1	1.32 ± 0.05	24.39 ± 0.05	Passable
PB2	1.33 ± 0.00	25.00 ± 0.00	Passable

**Table 4:** Mass and diameter of tablets obtained by SLS printing (mean ± SD) (printing parameters are given in the brackets in the following order: surface temperature (°C), chamber temperature (°C), and laser speed (mm/s)).

Tablet	Formulation	Diameter (mm)	Thickness (mm)	Weight (mg)
T1 (80 70 50)	PB 1	9.02 ± 0.01	3.73 ± 0.01	121.55 ± 1.57
T2 (80 70 60)		9.04 ± 0.01	3.74 ± 0.01	120.45 ± 2.14
T3 (90 80 50)		8.99 ± 0.03	3.70 ± 0.02	124.65 ± 1.18
T4 (90 80 60)		9.01 ± 0.01	3.71 ± 0.01	122.35 ± 1.27
T5 (80 70 50)	PB 2	8.96 ± 0.12	3.93 ± 0.14	158.67 ± 5.18
T6 (80 70 60)		8.99 ± 0.10	3.97 ± 0.10	142.91 ± 6.87
T7 (90 80 50)		8.92 ± 0.11	3.92 ± 0.09	168.25 ± 4.34
T8 (90 80 60)		8.98 ± 0.14	3.96 ± 0.11	153.88 ± 5.41

**Table 5:** Drug assay and uniformity of the content (mean ± SD).

Tablet	Amount of CVD (mg)	CVD Content (%) *	Uniformity of Content (AV(%)) **
T1 (80 70 50)	12.67 ± 0.12	104.28 ± 1.23	7.89 ± 0.77
T2 (80 70 60)	12.61± 0.14	104.73 ± 1.64	8.88 ± 0.90
T3 (90 80 50)	12.98 ± 0.21	104.17 ± 2,73	7.19 ± 1.03
T4 (90 80 60)	12.89 ± 0.11	105.39 ± 1.5	5.72 ± 0.45
T5 (80 70 50)	14.83 ± 0.13	93.44 ±0.90	7.58 ± 0.55
T6 (80 70 60)	14.69 ± 0.04	102.80 ± 0.05	6.44 ± 0. 43
T7 (90 80 50)	15.69 ± 0.16	93.28 ± 1.49	5.86 ± 0.68
T8 (90 80 60)	14.58 ± 0.14	94.74 ± 1.23	7.03 ± 0.49

* Acceptance criterion or CVD content: ±10% of the declared content (the United States Pharmacopeia–National Formulary 2024 (USP–NF 2024)–Carvedilol tablets). ** Acceptance criterion for Acceptance value (AV): <15% (European Pharmacopoeia 11th ed–Chapter 2.9.40).

**Table 6:** Hardness of SLS 3D-printed tablets (mean ± SD) (printing parameters are given in the brackets in the following order: surface temperature (°C), chamber temperature (°C), and laser speed (mm/s)).

Tablet	Formulation	Hardness (N)	Tensile Strength (MPa)
T1 (80 70 50)	PB 1	58.0 ± 1.0	1.16 ± 0.021
T2 (80 70 60)		54.33 ± 1.15	1.08 ± 0.024
T3 (90 80 50)		64.67 ± 0.58	1.31 ± 0.01
T4 (90 80 60)		60.33 ± 0.58	1.21 ± 0.013
T5 (80 70 50)	PB 2	62.5 ± 14.6	1.13 ± 0.29
T6 (80 70 60)		55.8 ± 16.2	0.97 ± 0.30
T7 (90 80 50)		70.8 ± 19.6	1.28 ± 0.37
T8 (90 80 60)		65.5 ± 18.1	1.19 ± 0.33

**Table 7:** Disintegration of SLS tablets obtained from powder blends 1 and 2 (printing parameters are given in the brackets in the following order: surface temperature (°C), chamber temperature (°C), and laser speed (mm/s)).

**PB 1**	**T1 (80 70 50)**	**T2 (80 70 60)**	**T3 (90 80 50)**	**T4 (90 80 60)**
	6 min 05 s	5 min 52 s	6 min 48 s	6 min 30 s
**PB 2**	**T5 (80 70 50)**	**T6 (80 70 60)**	**T7 (90 80 50)**	**T8 (90 80 60)**
	2 min 15 s	2 min 10 s	2 min 35 s	2 min 28 s

**Table 8:** Crystallinity of SLS 3D-printed tablets (printing parameters are given in the brackets in the following order: surface temperature (°C), chamber temperature (°C), and laser speed (mm/s)) and powders: carvedilol and mannitol.

Sample	Crystallinity (%)
T1 (80 70 50)	24.5
T2 (80 70 60)	25.4
T3 (90 80 50)	25.6
T4 (90 80 60)	27.3
T5 (80 70 50)	46.4
T6 (80 70 60)	47.9
T7 (90 80 50)	42.8
T8 (90 80 60)	44.1
Carvedilol	90.0
Mannitol	88.6

## Data Availability

The data presented in this study are available on request.

## References

[1] Awad A., Fina F., Goyanes A., Gaisford S., Basit A.W. (2020). 3D Printing: Principles and Pharmaceutical Applications of Selective Laser Sintering. Int. J. Pharm..

[2] Medarević D., Krstić M., Ibrić S. (2024). Fundamentals of 3D Printing of Pharmaceuticals. From Current to Future Trends in Pharmaceutical Technology.

[3] Chen G., Xu Y., Chi Lip Kwok P., Kang L. (2020). Pharmaceutical Applications of 3D Printing. Addit. Manuf..

[4] Pandey M., Choudhury H., Fern J.L.C., Kee A.T.K., Kou J., Jing J.L.J., Her H.C., Yong H.S., Ming H.C., Bhattamisra S.K. (2020). 3D Printing for Oral Drug Delivery: A New Tool to Customize Drug Delivery. Drug Deliv. Transl. Res..

[5] Wang S., Chen X., Han X., Hong X., Li X., Zhang H., Li M., Wang Z., Zheng A. (2023). A Review of 3D Printing Technology in Pharmaceutics: Technology and Applications, Now and Future. Pharmaceutics.

[6] Gueche Y.A., Sanchez-Ballester N.M., Cailleaux S., Bataille B., Soulairol I. (2021). Selective Laser Sintering (SLS), a New Chapter in the Production of Solid Oral Forms (SOFs) by 3D Printing. Pharmaceutics.

[7] Infanger S., Haemmerli A., Iliev S., Baier A., Stoyanov E., Quodbach J. (2019). Powder Bed 3D-Printing of Highly Loaded Drug Delivery Devices with Hydroxypropyl Cellulose as Solid Binder. Int. J. Pharm..

[8] Yan T.-T., Lv Z.-F., Tian P., Lin M.-M., Lin W., Huang S.-Y., Chen Y.-Z. (2020). Semi-Solid Extrusion 3D Printing ODFs: An Individual Drug Delivery System for Small Scale Pharmacy. Drug Dev. Ind. Pharm..

[9] Gao Y., Xu L., Zhao Y., You Z., Guan Q. (2020). 3D Printing Preview for Stereo-Lithography Based on Photopolymerization Kinetic Models. Bioact. Mater..

[10] Fina F., Goyanes A., Gaisford S., Basit A.W. (2017). Selective Laser Sintering (SLS) 3D Printing of Medicines. Int. J. Pharm..

[11] Adamov I., Stanojević G., Pavlović S.M., Medarević D., Ivković B., Kočović D., Ibrić S. (2024). Powder Bed Fusion–Laser Beam (PBF-LB) Three-Dimensional (3D) Printing: Influence of Laser Hatching Distance on the Properties of Zolpidem Tartrate Tablets. Int. J. Pharm..

[12] Goyanes A., Fina F., Martorana A., Sedough D., Gaisford S., Basit A.W. (2017). Development of Modified Release 3D Printed Tablets (Printlets) with Pharmaceutical Excipients Using Additive Manufacturing. Int. J. Pharm..

[13] Tabriz A.G., Gonot-Munck Q., Baudoux A., Garg V., Farnish R., Katsamenis O.L., Hui H.-W., Boersen N., Roberts S., Jones J. (2023). 3D Printing of Personalised Carvedilol Tablets Using Selective Laser Sintering. Pharmaceutics.

[14] Basit A.W. (2018). 3D Printing of Pharmaceuticals.

[15] Leong K.F., Chua C.K., Gui W.S. (2006). Verani Building Porous Biopolymeric Microstructures for Controlled Drug Delivery Devices Using Selective Laser Sintering. Int. J. Adv. Manuf. Technol..

[16] Awad A., Fina F., Goyanes A., Gaisford S., Basit A.W. (2021). Advances in Powder Bed Fusion 3D Printing in Drug Delivery and Healthcare. Adv. Drug Deliv. Rev..

[17] Yang Y., Xu Y., Wei S., Shan W. (2021). Oral Preparations with Tunable Dissolution Behavior Based on Selective Laser Sintering Technique. Int. J. Pharm..

[18] Vithani K., Goyanes A., Jannin V., Basit A.W., Gaisford S., Boyd B.J. (2019). An Overview of 3D Printing Technologies for Soft Materials and Potential Opportunities for Lipid-Based Drug Delivery Systems. Pharm. Res..

[19] Fina F., Madla C.M., Goyanes A., Zhang J., Gaisford S., Basit A.W. (2018). Fabricating 3D Printed Orally Disintegrating Printlets Using Selective Laser Sintering. Int. J. Pharm..

[20] Mohamed E.M., Barakh Ali S.F., Rahman Z., Dharani S., Ozkan T., Kuttolamadom M.A., Khan M.A. (2020). Formulation Optimization of Selective Laser Sintering 3D-Printed Tablets of Clindamycin Palmitate Hydrochloride by Response Surface Methodology. AAPS PharmSciTech.

[21] Aikawa S., Tanaka H., Ueda H., Maruyama M., Higaki K. (2022). Formation of a Stable Co-Amorphous System for a Brick Dust Molecule by Utilizing Sodium Taurocholate with High Glass Transition Temperature. Pharmaceutics.

[22] Liu J., Grohganz H., Löbmann K., Rades T., Hempel N.-J. (2021). Co-Amorphous Drug Formulations in Numbers: Recent Advances in Co-Amorphous Drug Formulations with Focus on Co-Formability, Molar Ratio, Preparation Methods, Physical Stability, In Vitro and In Vivo Performance, and New Formulation Strategies. Pharmaceutics.

[23] Eesam S., Bhandaru J.S., Naliganti C., Bobbala R.K., Akkinepally R.R. (2020). Solubility Enhancement of Carvedilol Using Drug–Drug Cocrystallization with Hydrochlorothiazide. Futur. J. Pharm. Sci..

[24] Johnson J.A., Cavallari L.H. (2013). Pharmacogenetics and Cardiovascular Disease—Implications for Personalized Medicine. Pharmacol. Rev..

[25] (2022). European Pharmacopoeia.

[26] Barakh Ali S.F., Mohamed E.M., Ozkan T., Kuttolamadom M.A., Khan M.A., Asadi A., Rahman Z. (2019). Understanding the Effects of Formulation and Process Variables on the Printlets Quality Manufactured by Selective Laser Sintering 3D Printing. Int. J. Pharm..

[27] (2024). The United States Pharmacopeia–National Formulary 2024 (USP–NF 2024).

[28] Vodáčková P., Vraníková B., Svačinová P., Franc A., Elbl J., Muselík J., Kubalák R., Solný T. (2018). Evaluation and Comparison of Three Types of Spray Dried Coprocessed Excipient Avicel^®^ for Direct Compression. BioMed Res. Int..

[29] Hiendrawan S., Widjojokusumo E., Veriansyah B., Tjandrawinata R.R. (2017). Pharmaceutical Salts of Carvedilol: Polymorphism and Physicochemical Properties. AAPS PharmSciTech.

[30] Alves J.M.V., Prado L.D., Rocha H.V.A. (2016). Evaluation and Correlation of the Physicochemical Properties of Carvedilol. Pharm. Dev. Technol..

[31] Medarević D., Djuriš J., Ibrić S., Mitrić M., Kachrimanis K. (2018). Optimization of Formulation and Process Parameters for the Production of Carvedilol Nanosuspension by Wet Media Milling. Int. J. Pharm..

[32] Guimarães T.F., Lanchote A.D., Da Costa J.S., Viçosa A.L., De Freitas L.A.P. (2015). A Multivariate Approach Applied to Quality on Particle Engineering of Spray-Dried Mannitol. Adv. Powder Technol..

[33] Altay Benetti A., Bianchera A., Buttini F., Bertocchi L., Bettini R. (2021). Mannitol Polymorphs as Carrier in DPIs Formulations: Isolation Characterization and Performance. Pharmaceutics.

[34] Madžarević M., Medarević Đ., Pavlović S., Ivković B., Đuriš J., Ibrić S. (2021). Understanding the Effect of Energy Density and Formulation Factors on the Printability and Characteristics of SLS Irbesartan Tablets—Application of the Decision Tree Model. Pharmaceutics.

[35] Prado L.D., Rocha H.V.A., Resende J.A.L.C., Ferreira G.B., De Figuereido Teixeira A.M.R. (2014). An Insight into Carvedilol Solid Forms: Effect of Supramolecular Interactions on the Dissolution Profiles. CrystEngComm.

[36] Andrade J., González-Martínez C., Chiralt A. (2020). The Incorporation of Carvacrol into Poly (Vinyl Alcohol) Films Encapsulated in Lecithin Liposomes. Polymers.

[37] Shamma R.N., Basha M. (2013). Soluplus^®^: A Novel Polymeric Solubilizer for Optimization of Carvedilol Solid Dispersions: Formulation Design and Effect of Method of Preparation. Powder Technol..

[38] Kor I., Wizel S. (2004). Crystalline Solids of Carvedilol and Processes for Their Preparation. U. S. Patent.

[39] Pešić N., Dapčević A., Ivković B., Kachrimanis K., Mitrić M., Ibrić S., Medarević D. (2021). Potential Application of Low Molecular Weight Excipients for Amorphization and Dissolution Enhancement of Carvedilol. Int. J. Pharm..

[40] Kaushal A.M., Gupta P., Bansal A.K. (2004). Amorphous Drug Delivery Systems: Molecular Aspects, Design, and Performance. Crit. Rev. Ther. Drug Carr. Syst..

[41] Wei Y., Dattachowdhury B., Vangara K.K., Patel N., Alexander K., Boddu S.H., Narang A.S., Boddu S.H.S. (2015). Excipients That Facilitate Amorphous Drug Stabilization. Excipient Applications in Formulation Design and Drug Delivery.

[42] Davis D.A., Thakkar R., Su Y., Williams R.O., Maniruzzaman M. (2021). Selective Laser Sintering 3-Dimensional Printing as a Single Step Process to Prepare Amorphous Solid Dispersion Dosage Forms for Improved Solubility and Dissolution Rate. J. Pharm. Sci..

[43] Trenfield S.J., Xu X., Goyanes A., Rowland M., Wilsdon D., Gaisford S., Basit A.W. (2023). Releasing Fast and Slow: Non-Destructive Prediction of Density and Drug Release from SLS 3D Printed Tablets Using NIR Spectroscopy. Int. J. Pharm. X.

